# Population immunity of natural infection, primary-series vaccination, and booster vaccination in Qatar during the COVID-19 pandemic: an observational study

**DOI:** 10.1016/j.eclinm.2023.102102

**Published:** 2023-07-20

**Authors:** Suelen H. Qassim, Hiam Chemaitelly, Houssein H. Ayoub, Peter Coyle, Patrick Tang, Hadi M. Yassine, Asmaa A. Al Thani, Hebah A. Al-Khatib, Mohammad R. Hasan, Zaina Al-Kanaani, Einas Al-Kuwari, Andrew Jeremijenko, Anvar Hassan Kaleeckal, Ali Nizar Latif, Riyazuddin Mohammad Shaik, Hanan F. Abdul-Rahim, Gheyath K. Nasrallah, Mohamed Ghaith Al-Kuwari, Adeel A. Butt, Hamad Eid Al-Romaihi, Mohamed H. Al-Thani, Abdullatif Al-Khal, Roberto Bertollini, Laith J. Abu-Raddad

**Affiliations:** a Infectious Disease Epidemiology Group, Weill Cornell Medicine-Qatar, Cornell University, Doha, Qatar; b World Health Organization Collaborating Centre for Disease Epidemiology Analytics on HIV/AIDS, Sexually Transmitted Infections, and Viral Hepatitis, Weill Cornell Medicine–Qatar, Cornell University, Qatar Foundation – Education City, Doha, Qatar; c Department of Population Health Sciences, Weill Cornell Medicine, Cornell University, New York, NY, USA; d Mathematics Program, Department of Mathematics, Statistics, and Physics, College of Arts and Sciences, Qatar University, Doha, Qatar; e Hamad Medical Corporation, Doha, Qatar; f Biomedical Research Center, Member of QU Health, Qatar University, Doha, Qatar; g Wellcome-Wolfson Institute for Experimental Medicine, Queens University, Belfast, United Kingdom; h Department of Pathology, Sidra Medicine, Doha, Qatar; i Department of Biomedical Science, College of Health Sciences, Member of QU Health, Qatar University, Doha, Qatar; j Department of Public Health, College of Health Sciences, QU Health, Qatar University, Doha, Qatar; k Primary Health Care Corporation, Doha, Qatar; l Department of Medicine, Weill Cornell Medicine, Cornell University, New York, NY, USA; m Ministry of Public Health, Doha, Qatar; n College of Health and Life Sciences, Hamad Bin Khalifa University, Doha, Qatar

**Keywords:** COVID-19, Immunity, Natural infection, Vaccine, Test-negative, Epidemiology

## Abstract

**Background:**

Waning of natural infection protection and vaccine protection highlight the need to evaluate changes in population immunity over time. Population immunity of previous SARS-CoV-2 infection or of COVID-19 vaccination are defined, respectively, as the overall protection against reinfection or against breakthrough infection at a given point in time in a given population.

**Methods:**

We estimated these population immunities in Qatar's population between July 1, 2020 and November 30, 2022, to discern generic features of the epidemiology of SARS-CoV-2. Effectiveness of previous infection, mRNA primary-series vaccination, and mRNA booster (third-dose) vaccination in preventing infection were estimated, month by month, using matched, test-negative, case–control studies.

**Findings:**

Previous-infection effectiveness against reinfection was strong before emergence of Omicron, but declined with time after a wave and rebounded after a new wave. Effectiveness dropped after Omicron emergence from 88.3% (95% CI: 84.8–91.0%) in November 2021 to 51.0% (95% CI: 48.3–53.6%) in December 2021. Primary-series effectiveness against infection was 84.0% (95% CI: 83.0–85.0%) in April 2021, soon after introduction of vaccination, before waning gradually to 52.7% (95% CI: 46.5–58.2%) by November 2021. Effectiveness declined linearly by ∼1 percentage point every 5 days. After Omicron emergence, effectiveness dropped from 52.7% (95% CI: 46.5–58.2%) in November 2021 to negligible levels in December 2021. Booster effectiveness dropped after Omicron emergence from 83.0% (95% CI: 65.6–91.6%) in November 2021 to 32.9% (95% CI: 26.7–38.5%) in December 2021, and continued to decline thereafter. Effectiveness of previous infection and vaccination against severe, critical, or fatal COVID-19 were generally >80% throughout the study duration.

**Interpretation:**

High population immunity against infection may not be sustained beyond a year, but population immunity against severe COVID-19 is durable with slow waning even after Omicron emergence.

**Funding:**

The Biomedical Research Program and the Biostatistics, Epidemiology, and the Biomathematics Research Core, both at 10.13039/100019460Weill Cornell Medicine-Qatar, 10.13039/501100004397Ministry of Public Health, 10.13039/100007833Hamad Medical Corporation, 10.13039/100019475Sidra Medicine, Qatar Genome Programme, Qatar University Biomedical Research Center, and Qatar University Internal Grant ID QUCG-CAS-23/24-114.

**Research in context**

**Evidence before this study**

SARS-CoV-2 infection induces protection against reinfection, but this protection wanes with time since last infection. Similarly, COVID-19 primary-series and booster vaccination induce protection against SARS-CoV-2 infection, but this protection also wanes with time since last dose. These immunity patterns demonstrate the need for the concept of *population immunity* to track evolution of overall immune protection over time in a given population. Previous-infection and vaccine population immunities in a specific country can be defined as the overall protection against infection at a given point in time in the full national population. A search of PubMed, Google Scholar, and the International Vaccine Access Center's VIEW-hub databases up to April 21, 2023 using the keywords “vaccination”, “infection”, “immunity”, “protection”, “SARS-CoV-2”, and “COVID-19” did not identify studies that investigated this epidemiological concept for a national population throughout the COVID-19 pandemic.

**Added value of this study**

This study analyzed the national federated databases for SARS-CoV-2 infection and COVID-19 vaccination in Qatar, a country that experienced SARS-CoV-2 waves dominated by different pre-Omicron variants and Omicron subvariants. Using a matched, test-negative study design, population immunity against infection of each of previous infection, primary-series vaccination, and booster vaccination were characterized at the national level month by month for two calendar years to discern generic features of the epidemiology of SARS-CoV-2. The three forms of population immunity showed rapid variation over time driven by waning of protection. Vaccine-derived population immunity declined by 1 absolute percentage point every 5 days. Omicron introduction immensely reduced the three forms of population immunity within one month by about 50 absolute percentage points. Meanwhile, previous-infection and vaccine population immunities against severe COVID-19 were durable with slow waning even after Omicron emergence.

**Implications of all the available evidence**

Both previous-infection and vaccine population immunities vary rapidly at a national level creating fertile grounds for repeated waves of infection to occur even within months of each other. High levels of population immunity may not be sustained for more than a year or so. Timely administration of boosters for those vulnerable to severe COVID-19 may remain essential for years to come. Emergence of a new variant that is substantially different from circulating variants can suddenly and immensely reduce population immunity leading to large epidemic waves. However, the durability of population immunity against severe COVID-19 will likely curtail the severity of future waves.

## Introduction

Although immune protection of the primary series of coronavirus disease 2019 (COVID-19) vaccines is high against severe acute respiratory syndrome coronavirus 2 (SARS-CoV-2) infection immediately after the second dose,^1^^,^^2^ protection wanes with time and may not last for more than 1 year after the second dose.^3^, ^4^, ^5^ The emergence of the immune-evasive Omicron subvariants reduced vaccine effectiveness immediately after the second dose to only about 50% and accelerated the waning of protection.^6^, ^7^, ^8^ Vaccine protection against Omicron subvariants may not last for more than 6 months after the second dose.^6^, ^7^, ^8^ Booster vaccination restores vaccine protection to the level observed immediately after the second dose,^8^^,^^9^ but this protection also wanes with time and in a similar pattern to that of the primary series.^6^, ^7^, ^8^, ^9^, ^10^

Though protection of natural infection against reinfection is high immediately after infection,^11^^,^^12^ the protection wanes with time and is not expected to last for more than 3 years.^13^ Introduction of Omicron subvariants reduced pre-Omicron infection protection against Omicron reinfection to only about 50%,^14^^,^^15^ and accelerated the waning of this protection.^13^ Protection of a pre-Omicron infection against Omicron reinfection may not last for more than 1 year.^13^^,^^16^ While protection of Omicron infection against Omicron reinfection is high,^16^, ^17^, ^18^ evidence suggests that this protection is declining fairly rapidly due to combined effect of waning of protection over time and progressive immune evasiveness of Omicron subvariants.^16^

These immune protection patterns demonstrate the need for the concept of *population immunity* to track evolution of population-level immune protection over time in a given population. It is important to distinguish this concept from the concepts of vaccine effectiveness since the last dose or protection against reinfection since the time of the last infection. These specific aspects have already been investigated in previous studies conducted in various countries, including within the population of Qatar, for different vaccines and age groups.^5^, ^6^, ^7^, ^8^, ^9^^,^^13^^,^^19^ However, merely knowing that vaccine effectiveness diminishes does not provide an understanding of the level of population immune protection at a given point in time or the timing of an upcoming wave, as individuals receive vaccinations at varying times. These distinct immune protection concepts complement each other, offer unique scientific insights, and generate various types of inferences.

Population immunity of primary-series vaccination is defined as the overall protection against infection at a given point in time among persons with only primary-series vaccination relative to those unvaccinated. Booster and previous-infection population immunities are defined analogously. These three forms of population immunity are expected to vary fairly rapidly even within a time horizon of only 1 year due to the combined effects of waning of vaccine and natural immunity and viral immune evasion. Such rapid variation may create fertile grounds for repeated waves of infection to occur even within few months of each other.

**Fig. 1:**
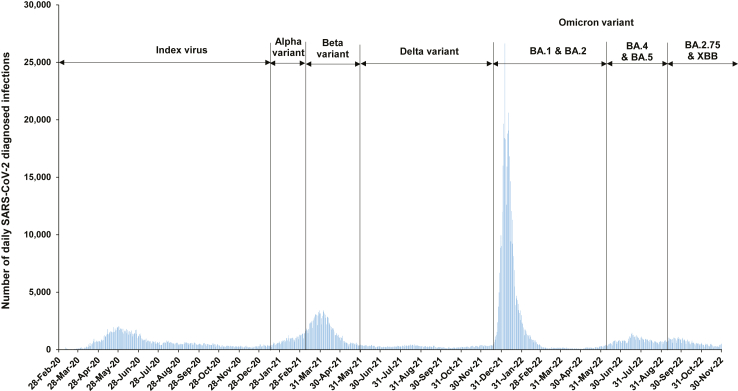
**Incidence of SARS-CoV-2 infection in Qatar**.

We aimed to characterize variation of these three forms of population immunity over time, month by month, in Qatar, a country that experienced SARS-CoV-2 waves dominated sequentially by the index virus,^20^ Alpha,^21^ Beta,^22^ Omicron BA.1 and BA.2,^14^ Omicron BA.4 and BA.5,^18^ Omicron BA.2.75∗,^16^ and currently Omicron XBB∗,^23^ in addition to a prolonged low-incidence phase dominated by Delta^24^ (Fig. 1).

## Methods

### Study population and data sources

This study was conducted on the population of Qatar including data between July 1, 2020 and November 30, 2022. It analyzed the national, federated databases for COVID-19 laboratory testing, vaccination, hospitalization, and death, retrieved from the integrated, nationwide, digital-health information platform (Section S1 of the Supplementary Appendix). Databases include all SARS-CoV-2-related data with no missing information since the onset of the pandemic, including all polymerase chain reaction (PCR) tests, and from January 5, 2022 onward, all medically supervised rapid antigen tests. SARS-CoV-2 testing in Qatar was done at large scale, and up to October 31, 2022, was mostly done for routine reasons such as for screening or travel-related purposes, with infections primarily diagnosed not because of appearance of symptoms, but because of routine testing.^3^^,^^14^ Qatar launched its COVID-19 vaccination program in December 2020 using almost exclusively the BNT162b2 and mRNA-1273 mRNAvaccines.^25^ By the conclusion of this study on November 30, 2021, 59.0% of individuals had received the BNT162b2 vaccine as part of their primary series vaccination, while 41.0% had received the mRNA-1273 vaccine. Detailed descriptions of Qatar's population and of the national databases have been reported previously.^3^^,^^9^^,^^10^^,^^14^^,^^20^^,^^26^

### Study design

Effectiveness of previous infection, two-dose (primary-series) vaccination, and third-dose (booster) vaccination in preventing infection, regardless of symptoms, were estimated for Qatar's population by calendar month. The reference group for comparing effectiveness consisted of individuals with no prior infection since the onset of the pandemic for evaluating the effectiveness of previous infection, and individuals with no COVID-19 vaccination since the onset of the pandemic for evaluating the effectiveness of vaccination. The calendar time over which these effectiveness measures were estimated depended on availability of primary-series and booster vaccination in the population.

Each of the effectiveness measures was estimated using the test-negative, case–control, study design, a standard design for assessing immune protection of natural infection^14^^,^^15^^,^^27^ and vaccination.^3^^,^^28^, ^29^, ^30^ In this design, effectiveness estimates are derived by comparing odds of previous infection or vaccination among positive tests (cases) compared to negative tests (controls).^27^, ^28^, ^29^

Effectiveness of previous infection in preventing reinfection was defined as the proportional reduction in susceptibility to infection among those with a previous infection versus those without.^12^^,^^14^^,^^27^ Effectiveness of primary-series (or booster) vaccination in preventing infection was defined as the proportional reduction in susceptibility to infection among those vaccinated versus those unvaccinated.^3^^,^^28^, ^29^, ^30^

All cases (SARS-CoV-2-positive tests) identified in each calendar month were exactly matched with controls (SARS-CoV-2-negative tests) on a one-to-one basis. The matching criteria included sex, 10-year age group, nationality, number of coexisting conditions (none, 1, 2, or ≥3), method of testing (PCR or rapid antigen), reason for SARS-CoV-2 testing, and the calendar month of testing (by design). The term “coexisting conditions” refers to chronic comorbidities, which were determined by examining the ICD-10 codes documented in the electronic health record encounters of each individual (Section S1).

Moreover, for analyses examining the effectiveness of previous infection, cases and controls were matched based on the COVID-19 vaccine type and number of vaccine doses at the time of SARS-CoV-2 testing. Similarly, for vaccine effectiveness analyses, matching was performed according to the status of the most recent prior infection (no documented prior infection since onset of the pandemic, documented pre-Omicron prior infection, or documented Omicron prior infection). This matching process was implemented to mitigate the influence of the immune protection that is not being assessed and to ensure comparability between the groups.

The numeric ratio of cases to controls varied throughout the study based on the incidence level, but generally, there were several times more controls than cases due to the extensive testing conducted. Approximately 5% of the population underwent testing every week, primarily for routine reasons.^3^^,^^14^ By utilizing exact one-to-one matching, the numeric ratio of matched cases to controls was always at 1. Cases and controls that did not match were not included in the analysis. In each calendar month analysis, cases and controls were used only once.

Matching was done to balance observed confounders between exposure groups that are related to risk of infection in Qatar.^20^^,^^31^, ^32^, ^33^, ^34^ Matching by the considered factors was informed by results of prior studies that used matching to control for differences in infection risk in Qatar, including test-negative, case–control studies.^2^, ^3^, ^4^^,^^25^^,^^35^ For each calendar month, only the first SARS-CoV-2-positive test for each case and the first SARS-CoV-2-negative test for each control were included. Persons qualified as controls if they had at least one SARS-CoV-2-negative test and no record of a SARS-CoV-2-positive test in the considered calendar month.

All persons who received a vaccine other than BNT162b2 or mRNA-1273, or who received a different mix of vaccines, were excluded. Individuals who received a fourth vaccine dose (second booster dose) prior to the study SARS-CoV-2 test were excluded. Bivalent boosters have only been recently introduced in Qatar and remain at low coverage,^23^ thus they were not included in this study. Tests occurring within 14 days of a second dose or 7 days of a third dose were excluded. SARS-CoV-2 tests conducted because of travel-related requirements were excluded.^3^^,^^4^ This specific exclusion was necessary due to the different travel testing protocols for vaccinated and unvaccinated individuals. Including these tests in the analysis may introduce bias into the estimates of vaccine effectiveness, as demonstrated in prior studies.^3^^,^^4^

Moreover, these inclusion and exclusion criteria were implemented to allow for build-up of immunity after vaccination,^1^^,^^9^ and to minimize other types of potential bias, as informed by earlier analyses on the same population.^3^^,^^4^ Every case (or control) that met the inclusion criteria and that could be matched to a control (case) was included in the analysis. Previous infection status and COVID-19 vaccination status were ascertained at the time of the SARS-CoV-2 test.

SARS-CoV-2 reinfection is conventionally defined as a documented infection ≥90 days after an earlier infection, to avoid misclassification of prolonged SARS-CoV-2 positivity as reinfection, if a shorter time interval is used.^36^^,^^37^ Previous infection was thus defined as a SARS-CoV-2-positive test ≥90 days before the study's SARS-CoV-2 test.^14^^,^^15^^,^^27^ Cases or controls with SARS-CoV-2-positive tests <90 days before the study's SARS-CoV-2 test were excluded. Consequently, we were unable to commence the analyses in April 2020, when a significant incidence of infection was first observed. We had to wait for the 90-day period to elapse, which led us to initiate the analyses in July 2020, enabling a distinction of reinfections.

In relation to vaccination, the rollout of the first dose commenced in late December 2020. However, by the end of January 2021, only a limited number of individuals had completed the primary series of vaccination. To ensure an adequate sample size and representative data, we initiated the vaccination analyses in February 2021.

Similarly, for the third-dose analyses, we commenced the investigations in November 2021, taking into account similar considerations of sample size and representation.

In the analyses examining the effectiveness of previous infection, the study included a total of 899,441 cases and 10,709,791 controls throughout all months. The matched samples in the study consisted of 530,213 cases and 530,213 controls over the entire duration.

In the analyses examining the effectiveness of two-dose (primary-series) vaccination, the study included a total of 796,255 cases and 9,126,914 controls throughout all months. The matched samples in the study consisted of 382,978 cases and 382,978 controls over the entire duration.

In the analyses examining the effectiveness of third-dose (booster) vaccination, the study included a total of 568,661 cases and 5,342,301 controls throughout all months. The matched samples in the study consisted of 156,115 cases and 156,115 controls over the entire duration.

Effectiveness was also estimated against severe, critical, or fatal COVID-19 due to SARS-CoV-2 infection using the same methodology, but for longer calendar-month durations (instead of one-month duration) owing to the small number of cases with severe forms of COVID-19. Cases and controls were matched one-to-five to increase precision of estimates. Classification of COVID-19 case severity (acute-care hospitalizations),^38^ criticality (intensive-care-unit hospitalizations),^38^ and fatality^39^ followed World Health Organization (WHO) guidelines, and assessments were made by trained medical personnel using individual chart reviews (Section S3).

Each person who had a SARS-CoV-2-positive test and COVID-19 hospital admission was subject to an infection severity assessment every three days until discharge or death, regardless of the length of hospital stay or the time between the SARS-CoV-2-positive test and the final disease outcome. Individuals who progressed to severe,^38^ critical,^38^ or fatal^39^ COVID-19 between the SARS-CoV-2-positive test and the end of study were classified based on their worst outcome, starting with death, followed by critical disease, and then severe disease.

### Oversight

The institutional review boards at Hamad Medical Corporation and Weill Cornell Medicine–Qatar approved this retrospective study with a waiver of informed consent. The study was reported according to the Strengthening the Reporting of Observational Studies in Epidemiology (STROBE) guidelines (STROBE checklist).

### Statistical analysis

While all records of SARS-CoV-2 testing were examined for selection of cases and controls, only matched samples were analyzed. Cases and controls were described using frequency distributions and measures of central tendency and compared using standardized mean differences (SMDs). An SMD of ≤0.1 indicated adequate matching.^40^ The median and interquartile range (IQR) of the duration between the immunological event (previous infection, primary-series vaccination, or booster vaccination) and SARS-CoV-2 test were calculated for cases and controls in each calendar-month analysis.

The odds ratio (and its associated 95% confidence interval (CI)), comparing odds of the immunological event among cases to that among controls, was estimated using conditional logistic regression, that is factoring the matching in the study design. This analytical approach was implemented to reduce potential bias due to variation in epidemic phase,^28^^,^^41^ gradual vaccination roll-out,^28^^,^^41^ and other confounders.^20^^,^^42^^,^^43^ CIs did not factor multiplicity and interactions were not examined.

Vaccine effectiveness and associated 95% CI were estimated as 1-odds ratio (OR) of the immunological event among cases versus controls if the OR was <1,^27^, ^28^, ^29^ and as (1/OR)-1 if the OR was ≥1.^10^^,^^44^ The latter was done to ensure symmetric scale for both negative and positive effectiveness, ranging from −100% to 100%.^10^^,^^44^ Statistical analyses were conducted in STATA/SE version 17.0 (Stata Corporation, College Station, TX, USA).

### Role of the funding source

The funders of the study had no role in study design, data collection, data analysis, data interpretation, or writing of the report. The corresponding authors had full access to all the data in the study and had final responsibility for the decision to submit for publication.

## Results

### Effectiveness of previous infection against reinfection

**Fig. 2:**
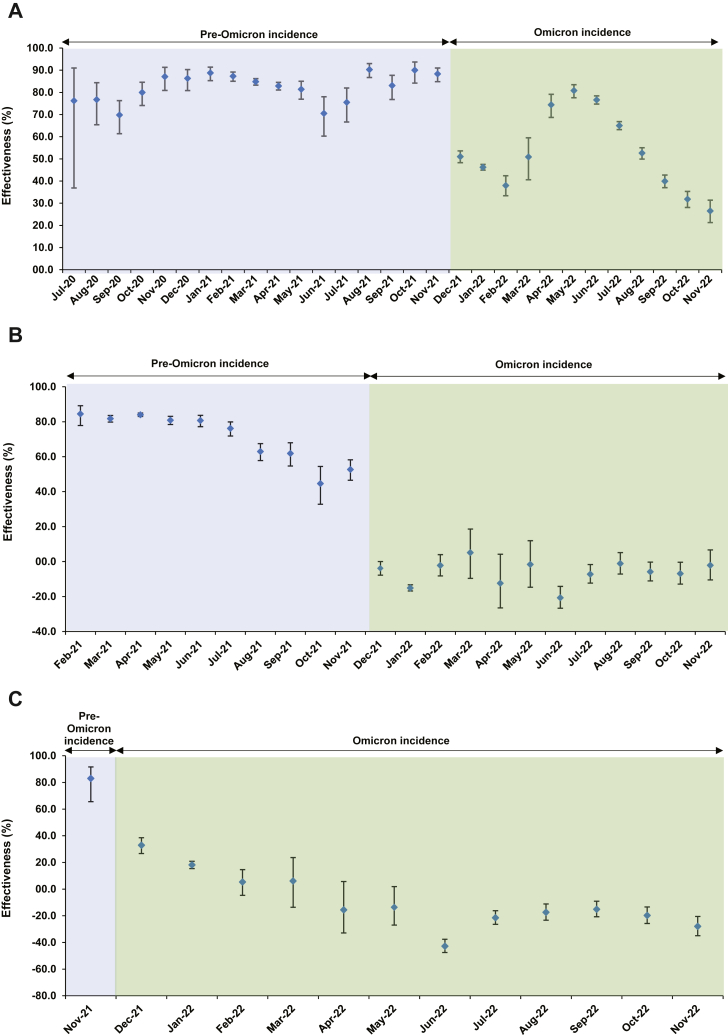
**Effectiveness of A) previous SARS-CoV-2 infection in preventing reinfection, B) primary-series (two-dose) mRNA vaccination in preventing infection, and C) booster (third-dose) mRNA vaccination in preventing infection, in Qatar, between July 2020 and November 2022∗**. ^∗^Most SARS-CoV-2 infections diagnosed in December 2021 were Omicron infections, whereas a minority were due to the Delta variant.

Characteristics of matched cases and controls of the study population are shown in Table S1. Figure S1 illustrates the selection process for the study population. Effectiveness of previous infection against reinfection was high from July 2020 to November 2021, a duration coinciding with incidence of pre-Omicron variants (Fig. 1), at a level that exceeded 70% (Table S2 and Fig. 2A). Effectiveness declined slowly over time after a wave, but rebounded to a higher level after a new wave, reflecting the recent increase in the number of individuals who were infected and protected against reinfection. Note that since cases and controls with SARS-CoV-2-positive tests <90 days before the study's SARS-CoV-2 test were excluded to avoid misclassification of prolonged SARS-CoV-2 positivity as reinfection,^36^^,^^37^ estimates of the effectiveness of previous infections represent infections that occurred ≥90 days prior and not that of very recent infections.

Effectiveness dropped immediately and immensely with the emergence of Omicron in December 2021 (Table S2 and Fig. 2A). While effectiveness was 88.3% (95% CI: 84.8–91.0%) in November 2021, it dropped to 51.0% (95% CI: 48.3–53.6%) in December 2021; a drop of ∼40 absolute percentage points within one month associated with the change in the circulating variant. Effectiveness continued at this level during the large BA.1/BA.2 Omicron wave, up to March 2022.

Effectiveness rebounded again after end of the BA.1/BA.2 wave and reached 80.8% (95% CI: 77.6–83.5%) in May 2022 before gradually declining in subsequent months (Table S2 and Fig. 2A). Effectiveness was low at ∼30% by end of the study in November 2022, a time coinciding with incidence of BA.2.75∗ and XBB∗ subvariants (Fig. 1).

### Effectiveness of primary-series vaccination against infection

Characteristics of matched cases and controls of the study population are shown in Table S1. Figure S2 illustrates the selection process for the study population. Effectiveness of primary-series vaccination against infection was very high at >80% right after introduction of vaccination (Table S3 and Fig. 2B). Effectiveness was 84.5% (95% CI: 77.8–89.2%) in February 2021 and remained high up to June 2021, coinciding with the rapid scale-up of vaccination in Qatar^3^ and incidence of pre-Omicron variants (Fig. 1).

However, starting from July 2021, when the mass vaccination campaigns slowed down along with a decline in the recentness of the second dose for much of the population, effectiveness started to decline rapidly (Table S3 and Fig. 2B). Effectiveness was 80.7% (95% CI: 77.2–83.7%) in June 2021 before dropping to 76.2% (95% CI: 71.8–79.9%) in July 2021, 63.0% (95% CI: 57.8–67.5%) in August 2021, 61.9% (95% CI: 54.7–68.0%) in September 2021, 44.6% (95% CI: 32.8–54.4%) in October 2021, and 52.7% (95% CI: 46.5–58.2%) in November 2021, right before introduction of Omicron (Fig. 1).

**Fig. 3:**
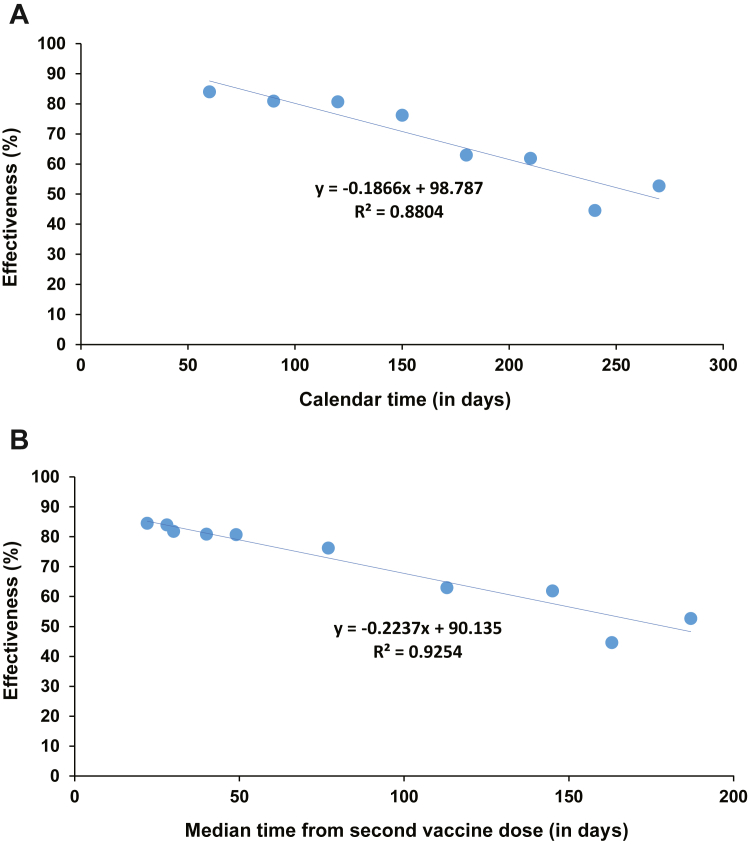
**Association between the effectiveness (per calendar month) of primary-series mRNA vaccination in preventing infection and (A) calendar time (in days) and (B) median time from second vaccine dose (in days)**.

Effectiveness declined linearly with calendar time by ∼1 absolute percentage point every 5 days (Fig. 3A). Effectiveness declined linearly with median time since the second dose also by ∼1 absolute percentage point every 5 days (Fig. 3B). Extrapolating this linear trend indicated that effectiveness would reach 0% in 13.4 months after the second dose.

Effectiveness dropped suddenly and massively with introduction of Omicron in December 2021 (Table S3 and Fig. 2B). While effectiveness was 52.7% (95% CI: 46.5–58.2%) in November 2021, it became negligible during the BA.1/BA.2 wave starting from December 2021, that is a drop of ∼50 absolute percentage points within one month associated with the change in circulating variant. Effectiveness remained negligible in subsequent months and was notably negative during the BA.4/BA.5 wave in June and July 2022.

### Effectiveness of booster vaccination against infection

Characteristics of matched cases and controls of the study population are shown in Table S1. Figure S3 illustrates the selection process for the study population. Effectiveness of booster (third dose) vaccination against infection was very high at 83.0% (95% CI: 65.6–91.6%) in November 2021 (Table S4 and Fig. 2C), when booster vaccination was being scaled up^9^ and Delta was circulating (Fig. 1). But effectiveness dropped suddenly and immensely with the emergence of Omicron in December 2021. Effectiveness dropped from 83.0% (95% CI: 65.6–91.6%) in November 2021 to 32.9% (95% CI: 26.7–38.5%) in December 2021, that is a drop of ∼50 absolute percentage points within one month associated with the change in the circulating variant. Effectiveness continued to decline thereafter and became negative between April and November 2022, particularly during the BA.4/BA.5 wave.

### Effectiveness against severe, critical, or fatal COVID-19

**Fig. 4:**
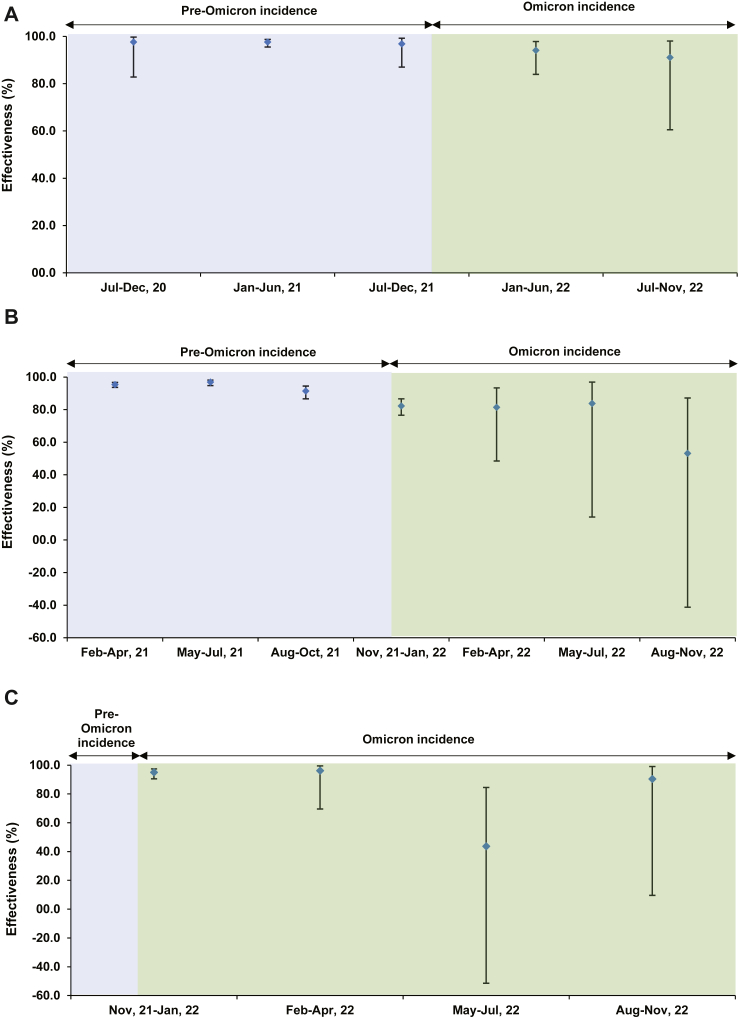
**Effectiveness against severe, critical, or fatal COVID-19 of A) previous SARS-CoV-2 infection, B) primary-series (two-dose) mRNA vaccination, and C) booster (three-dose) mRNA vaccination, in Qatar, between July 2020 and November 2022∗**. ^∗^Most SARS-CoV-2 infections diagnosed in December 2021 were Omicron infections, whereas a minority were due to the Delta variant.

Unlike effectiveness against infection, effectiveness against severe, critical, or fatal COVID-19 of previous infection (Table S5 and Fig. 4A), primary-series vaccination (Table S5 and Fig. 4B), and booster vaccination (Table S5 and Fig. 4C) were high throughout the study duration at generally >80%.

There was an indication of some decline in effectiveness of primary-series vaccination and booster vaccination over time; however, the limited number of severe COVID-19 cases hindered precise estimation of the potential waning in effectiveness. There were 12,227 cases of severe, critical, or fatal COVID-19 among 430,192 documented infections prior to the emergence of Omicron. After the emergence of Omicron, there were 525 cases of severe, critical, or fatal COVID-19 among 539,781 documented infections. These numbers underscore the relatively low severity of this pandemic within the young population of Qatar and the lower severity after Omicron emergence.^45^, ^46^, ^47^

## Discussion

This study investigated three population immunity patterns of relevance to the future of this pandemic. First, both natural and vaccine population immunity wane with time, with the waning being particularly rapid for vaccine population immunity. Even before Omicron introduction, vaccine protection was waning by ∼1 absolute percentage point every 5 days. The protection induced by vaccination against infection, symptomatic or asymptomatic, appeared to fully wane within 14 months. The decline in effectiveness observed prior to the emergence of Omicron appears primarily to result from the impact of waning immunity rather than the effect of the variant, confirming earlier findings.^3^^,^^4^^,^^24^ These findings suggest that repeated waves of infection will become a generic feature of SARS-CoV-2 epidemiology. High levels of population immunity may not be sustained for more than a year or so.

Second, sudden emergence of a new variant that is substantially different from previously circulating variants can immensely and immediately affect population immunity. Different variants emerged throughout the duration of the study; however, none of these variants displayed the abrupt and substantial level of immune evasion observed in the case of the Omicron variant. Protection of previous infection dropped by ∼40 absolute percentage points within only one month after Omicron introduction. Similarly, each of primary-series and booster protections dropped by ∼50 absolute percentage points. As a result, Qatar experienced its largest SARS-CoV-2 epidemic wave to date (Fig. 1). This demonstrates how the long-term epidemiology of this infection can be strongly sensitive to major shifts in virus evolution. Active surveillance of emerging variants and development of an early warning system are critical to mitigate the consequences of immune-evasive variants.^48^

Third, although population immunity against infection waned rapidly, population immunity against severe COVID-19 was durable over the study duration and showed slow waning even with introduction of Omicron. This slow waning appeared also to affect only vaccine immunity. The extent to which strong protection against severe COVID-19 will persist as more time elapses remains to be determined.

Infection with common-cold coronaviruses,^49^ and perhaps influenza,^50^ induces only a year-long protection against infection, but life-long immunity against severe reinfection.^51^ While it is premature to make long-term predictions, this finding suggests that SARS-CoV-2 epidemiology may exhibit a similar pattern to that of common-cold coronaviruses. Long-term immune protection against severe COVID-19 could contribute to a benign pattern of infection that is perhaps not dissimilar to that of common-cold coronaviruses.

Some of the vaccine effectiveness measures post Omicron introduction, particularly for booster vaccination, were negative in value, perhaps suggesting negative immune imprinting. This effect was pronounced during the BA.4/BA.5 wave. This finding supports similar recent findings in this same population.^10^^,^^52^ Such imprinting effects have been observed for other infections such as influenza.^53^^,^^54^ It remains to be seen whether these effects will be of consequence in the future epidemiology of SARS-CoV-2 infection.

This study has limitations. Since SARS-CoV-2 reinfection is defined as an infection ≥90 days after an earlier infection, estimates of previous-infection protection lagged actual level of protection by 3 months. This has underestimated previous-infection population immunity during or right after large waves, when population immunity was building up rapidly. With the relatively young population of Qatar, our findings may not be generalizable to other countries where elderly citizens constitute a larger proportion of the total population.

At this stage of the pandemic, the reference groups of persons with no previous infection and unvaccinated persons may not be representative due to depletion of susceptibles and undocumented infections and vaccinations. Vaccinations outside of Qatar may not have been registered in the national database after easing vaccine requirements in 2021. Infection ascertainment might have been influenced by changes in health-seeking and testing behaviors related to immune status over time. Bias can arise in real-world observational data in unexpected ways, or from unknown sources, including subtle differences in test-seeking behavior or changes in the pattern of testing due to policy changes, tests’ accessibility, or behavioral differences (Section S1). The reason for testing varies with time even if there are no policy changes. During a wave, a large proportion of testing occurs because of clinical suspicion of infection. At times of low incidence, a large proportion of testing occurs because of routine reasons.

However, until October 31, 2022, which is towards the end of the study period, the majority of tests conducted in Qatar were for routine purposes rather than due to the presence of symptoms.^3^^,^^14^ Approximately 5% of the population underwent testing every week, and around 75% of those diagnosed were identified through routine testing rather than symptom-based testing.^3^^,^^14^ This suggests that the ascertainment of infections may have remained consistent throughout the study.

To mitigate any potential bias resulting from shifts in health-seeking and testing behaviors influenced by immune status, we employed matching by vaccine type and number of doses in our analyses to evaluate the effectiveness of previous infection. Similarly, in the vaccine effectiveness analyses, matching was employed based on the status of the most recent prior infection.

Importantly, we have previously conducted mathematical modeling and statistical sensitivity analyses to assess the potential impact of misclassification bias related to immune-status, and these analyses supported the reliability of the test-negative design used in the present study.^3^^,^^27^ The odds ratio, the statistical metric utilized to estimate the effect size, often adjusts for the effects of misclassification bias, as this bias generally affects both the numerator and denominator of the odds ratio in a similar manner.^3^^,^^27^ In fact, this characteristic is recognized as one of the primary strengths of the test-negative case–control design.^3^^,^^27^

Having said so, bias due to depletion of susceptibles may still lead to underestimation of previous-infection or vaccine protections,^55^ even in the test-negative study design which is less prone to effect of this bias.^3^^,^^27^ This is especially the case if the majority of the population had already been infected.^27^ This source of bias may explain some of the negative vaccine effectiveness values observed in this study post Omicron introduction and the lower than expected previous-infection protection in the last few months of the study. Estimates after the first large Omicron wave may provide qualitative findings rather than precise quantitative estimates.

The study did not investigate population immunity of a second booster dose or of bivalent boosters due to the low coverage of these vaccinations in Qatar. In the first few weeks of the pandemic, PCR workflows were still under development and there was a chance of false-positive tests. While this may have affected only a very small number of PCR tests, reinfections were so rare at this time that few such false-positive cases could have resulted in underestimation of previous-infection protection in the first few months of the pandemic.

While matching was done for several factors, it was not possible for other factors such as geography or occupation, as such data were unavailable. However, Qatar is essentially a city state and infection incidence was broadly distributed across neighborhoods. Nationality, age, and sex provide a powerful proxy for socio-economic status in this country,^20^^,^^31^, ^32^, ^33^, ^34^ and thus matching by these factors may have partially controlled for other factors such as occupation. The matching prescription had already been investigated in previous studies of different epidemiologic designs, and using control groups to test for null effects.^2^, ^3^, ^4^^,^^25^^,^^35^ These studies have supported that this prescription provides adequate control of the differences in infection exposure.^2^, ^3^, ^4^^,^^25^^,^^35^ The study was implemented on Qatar's total population, perhaps minimizing the likelihood of bias.

In conclusion, both previous-infection and vaccine population immunities wane with time and the waning is rapid for vaccine protection. High levels of population immunity may not be sustained for more than a year. The waning of population immunity facilitates fertile grounds for repeated waves of infection to occur even within few months of each other. Timely administration of boosters for those vulnerable to COVID-19 may remain essential for years to come. Emergence of a new variant that is substantially different from previously circulating variants can suddenly and immensely reduce population immunity leading to large epidemic waves. Repeated waves of infection may also facilitate further evolution of the virus and continual immune evasion. However, with the durability of population immunity against severe COVID-19, the repeated epidemic waves may increasingly exhibit an overall benign pattern of infection.

## Contributors

SHQ, HC, and LJA co-designed the study. SHQ performed the statistical analyses and co-wrote the first draft of the article. LJA conceived the study, led the statistical analyses, and co-wrote the first draft of the article. SHQ, HC, and LJA accessed and verified all the data. PVC designed mass PCR testing to allow routine capture of SGTF variants and conducted viral genome sequencing. PT and MRH designed and conducted multiplex, RT-qPCR variant screening and viral genome sequencing. HY, AAA-T, and HAA-K conducted viral genome sequencing. All authors (SHQ, HC, HHA, PC, PT, HMY, AAA-T, HAA-K, MRH, ZA-K, EA-K, AJ, AHK, ANL, RMS, HFA-R, GKN, MGA-K, AAB, HEA-R, MHA-T, AA-K, RB, LJA) contributed to data collection and acquisition, database development, discussion and interpretation of the results, and to the writing of the manuscript. All authors have read and approved the final manuscript. Decision to publish the paper was by consensus among all authors.

## Data sharing statement

The dataset of this study is a property of the Qatar Ministry of Public Health that was provided to the researchers through a restricted-access agreement that prevents sharing the dataset with a third party or publicly. The data are available under restricted access for preservation of confidentiality of patient data. Access can be obtained through a direct application for data access to Her Excellency the Minister of Public Health (https://www.moph.gov.qa/english/OurServices/eservices/Pages/Governmental-HealthCommunication-Center.aspx). The raw data are protected and are not available due to data privacy laws. Aggregate data are available within the paper and its supplementary information.

## Declaration of interests

We declare no competing interests.

## Appendix A Supplementary data

Supplementary Appendix

STROBE checklist
