# Supplementary material for: Population immunity of natural infection, primary-series vaccination, and booster vaccination in Qatar during the COVID-19 pandemic: an observational study
Source: eClinicalMedicine. 2023 Jul 20;62:102102. doi: 10.1016/j.eclinm.2023.102102 (PMC10393554; doi:10.1016/j.eclinm.2023.102102)
Supplement: Supplementary Appendix [file mmc1.docx]

**Supplementary Appendix**

**Table of Contents**

[***Section S1*: Further details on methods** 2](#_Toc137686455)

[**Data sources and testing** 2](#_Toc137686456)

[**Comorbidity classification** 4](#_Toc137686457)

[***Section S2*: Laboratory methods and variant ascertainment** 6](#_Toc137686458)

[**Real-time reverse-transcription polymerase chain reaction testing** 6](#_Toc137686459)

[**Rapid antigen testing** 6](#_Toc137686460)

[**Classification of infections by variant type** 7](#_Toc137686461)

[***Section S3*: COVID-19 severity, criticality, and fatality classification** 8](#_Toc137686462)

[***Table S1*: Characteristics of matched cases and controls using the combined samples over all months of the studies investigating effectiveness against SARS-CoV-2 infection of previous SARS-CoV-2 infection, primary-series (two-dose) mRNA vaccination, and booster (third dose) mRNA vaccination.** 10](#_Toc137686463)

[***Figure S1:* Flowchart illustrating the population selection process to investigate the effectiveness of previous SARS-CoV-2 infection against reinfection in Qatar in a given calendar month analysis.** 11](#_Toc137686464)

[***Table S2*: Effectiveness of previous SARS-CoV-2 infection against reinfection in Qatar between July of 2020 and November of 2022.** 12](#_Toc137686465)

[***Figure S2:* Flowchart illustrating the population selection process to investigate the effectiveness of primary-series (two-dose) mRNA vaccination against SARS-CoV-2 infection in Qatar in a given calendar month analysis.** 13](#_Toc137686466)

[***Table S3:* Effectiveness of primary-series (two-dose) mRNA vaccination against SARS-CoV-2 infection in Qatar between February of 2021 and November of 2022.** 14](#_Toc137686467)

[***Figure S3:* Flowchart illustrating the population selection process to investigate the effectiveness of booster (third-dose) mRNA vaccination against SARS-CoV-2 infection in Qatar in a given calendar month analysis.** 15](#_Toc137686468)

[***Table S4:* Effectiveness of booster (third-dose) mRNA vaccination against SARS-CoV-2 infection in Qatar between November of 2021 and November of 2022.** 16](#_Toc137686469)

[***Table S5:* Effectiveness against severe, critical, or fatal COVID-19 of A) previous SARS-CoV-2 infection, B) primary-series (two-dose) mRNA vaccination, and C) booster (third-dose) mRNA vaccination, in Qatar, between July of 2020 and November of 2022.** 17](#_Toc137686470)

[***References*** 19](#_Toc137686471)

# ***Section S1*: Further details on** **methods**

## **Data sources and testing**

Qatar’s national and universal public healthcare system uses the Cerner-system advanced digital health platform to track all electronic health record encounters of each individual in the country, including all citizens and residents registered in the national and universal public healthcare system. Registration in the public healthcare system is mandatory for citizens and residents.

The databases analyzed in this study are data-extract downloads from the Cerner-system that have been implemented on a regular (twice weekly) schedule since the onset of the pandemic by the Business Intelligence Unit at Hamad Medical Corporation. Hamad Medical Corporation (HMC) is the national public healthcare provider in Qatar. At every download all tests, coronavirus disease 2019 (COVID-19) vaccinations, hospitalizations related to COVID-19, and all death records regardless of cause are provided to the authors through .csv files. These databases have been analyzed throughout the pandemic not only for study-related purposes, but also to provide policymakers with summary data and analytics to inform the national response.

Every health encounter in the Cerner-system is linked to a unique individual through the HMC Number that links all records for this individual at the national level. Databases were merged and analyzed using the HMC Number to link all records whether for testing, vaccinations, hospitalizations, and deaths. All deaths in Qatar are tracked by the public healthcare system. All COVID-19-related healthcare was provided only in the public healthcare system. No private entity was permitted to provide COVID-19-related healthcare. COVID-19 vaccination was also provided only through the public healthcare system. These health records were tracked throughout the COVID-19 pandemic using the Cerner system. This system has been implemented in 2013, before the onset of the pandemic.

Demographic details for every HMC Number (individual) such as sex, age, and nationality are collected upon issuing of the universal health card, based on the Qatar Identity Card, which is a mandatory requirement by the Ministry of Interior to every citizen and resident in the country.

Severe acute respiratory syndrome coronavirus 2 (SARS-CoV-2) testing in the healthcare system in Qatar is done at a mass scale, and up to October 31, 2022, was mostly done for routine reasons, where about 5% of the population were tested every week.^1,2^ All SARS-CoV-2 testing in any facility in this country is tracked nationally in one database, the national testing database. This database covers all testing in all locations and facilities throughout the country, whether public or private. Every polymerase chain reaction (PCR) test and an increasing proportion of the medically supervised rapid antigen tests conducted in Qatar, regardless of location or setting, are classified on the basis of symptoms and the reason for testing (clinical symptoms, contact tracing, surveys or random testing campaigns, individual requests, routine healthcare testing, pre-travel, at port of entry, or other). Based on the distribution of the reason for testing up to October 31, 2022, most of the tests that have been conducted in Qatar were conducted for routine reasons, such as being travel-related. About 75% of those diagnosed are also diagnosed not because of appearance of symptoms, but because of routine testing.^1,2^

The first large Omicron wave that peaked in January of 2022 was massive and strained the testing capacity in the country.^1,3^ Accordingly, rapid antigen testing was introduced to relieve the pressure on PCR testing. Implementation of this change in testing occurred quickly precluding incorporation of reason for testing in a large proportion of the rapid antigen tests for several months. While the reason for testing is available for all PCR tests, it is not available for all rapid antigen tests. Availability of reason for testing for the rapid antigen tests also varied with time.

Rapid antigen test kits are available for purchase in pharmacies in Qatar, but outcome of home-based testing is not reported nor documented in the national databases. Since SARS-CoV-2-test outcomes are linked to specific public health measures, restrictions, and privileges, testing policy and guidelines stress facility-based testing as the core testing mechanism in the population. While facility-based testing is provided free of charge or at low subsidized costs, depending on the reason for testing, home-based rapid antigen testing is de-emphasized and not supported as part of national policy.

Qatar has unusually young, diverse demographics, in that only 9% of its residents are ≥50 years of age, and 89% are expatriates from over 150 countries.^4,5^ Further descriptions of the study population and these national databases were reported previously.^1,2,5-8^

## **Comorbidity classification**

Comorbidities were ascertained and classified based on the ICD-10 codes as recorded in the electronic health record encounters of each individual in the Cerner-system national database that includes all citizens and residents registered in the national and universal public healthcare system. The public healthcare system provides healthcare to the entire resident population of Qatar free of charge or at heavily subsidized costs, including prescription drugs.

All encounters for each individual were analyzed to determine the comorbidity classification for that individual, as part of a recent national analysis to assess healthcare needs and resource allocation. The Cerner-system national database includes encounters starting from 2013, after this system was launched in Qatar. As long as each individual had at least one encounter with a specific comorbidity diagnosis since 2013, this person was classified with this comorbidity.

Individuals who have comorbidities but never sought care in the public healthcare system, or seek care exclusively in private healthcare facilities, were classified as individuals with no comorbidity due to absence of recorded encounters for them.

# ***Section S2*: Laboratory methods and variant ascertainment**

## **Real-time reverse-transcription polymerase chain reaction testing**

Nasopharyngeal and/or oropharyngeal swabs were collected for polymerase chain reaction (PCR) testing and placed in Universal Transport Medium (UTM). Aliquots of UTM were: 1) extracted on KingFisher Flex (Thermo Fisher Scientific, USA), MGISP-960 (MGI, China), or ExiPrep 96 Lite (Bioneer, South Korea) followed by testing with real-time reverse-transcription PCR (RT-qPCR) using TaqPath COVID-19 Combo Kits (Thermo Fisher Scientific, USA) on an ABI 7500 FAST (Thermo Fisher Scientific, USA); 2) tested directly on the Cepheid GeneXpert system using the Xpert Xpress SARS-CoV-2 (Cepheid, USA); or 3) loaded directly into a Roche cobas 6800 system and assayed with the cobas SARS-CoV-2 Test (Roche, Switzerland). The first assay targets the viral S, N, and ORF1ab gene regions. The second targets the viral N and E-gene regions, and the third targets the ORF1ab and E-gene regions.

All PCR testing was conducted at the Hamad Medical Corporation Central Laboratory or Sidra Medicine Laboratory, following standardized protocols.

## **Rapid antigen testing**

SARS-CoV-2 antigen tests were performed on nasopharyngeal swabs using one of the following lateral flow antigen tests: Panbio COVID-19 Ag Rapid Test Device (Abbott, USA); SARS-CoV-2 Rapid Antigen Test (Roche, Switzerland); Standard Q COVID-19 Antigen Test (SD Biosensor, Korea); or CareStart COVID-19 Antigen Test (Access Bio, USA). All antigen tests were performed point-of-care according to each manufacturer’s instructions at public or private hospitals and clinics throughout Qatar with prior authorization and training by the Ministry of Public Health (MOPH). Antigen test results were electronically reported to the MOPH in real time using the Antigen Test Management System which is integrated with the national Coronavirus Disease 2019 (COVID-19) database.

## **Classification of infections by variant type**

Surveillance for SARS-CoV-2 variants in Qatar is based on viral genome sequencing and multiplex RT-qPCR variant screening^9^ of random positive clinical samples,^2,10-14^ complemented by deep sequencing of wastewater samples.^12,15,16^ Further details on the viral genome sequencing and multiplex RT-qPCR variant screening throughout the SARS-CoV-2 waves in Qatar can be found in previous publications.^1-3,6,10-14,17-21^

# ***Section S3*: COVID-19 severity, criticality, and fatality classification**

Classification of COVID-19 case severity (acute-care hospitalizations),^22^ criticality (intensive-care-unit hospitalizations),^22^ and fatality^23^ followed World Health Organization (WHO) guidelines. Assessments were made by trained medical personnel independent of study investigators and using individual chart reviews, as part of a national protocol applied to every hospitalized COVID-19 patient. Each hospitalized COVID-19 patient underwent an infection severity assessment every three days until discharge or death. We classified individuals who progressed to severe, critical, or fatal COVID-19 between the time of the documented infection and the end of the study based on their worst outcome, starting with death,^23^ followed by critical disease,^22^ and then severe disease.^22^

Severe COVID-19 disease was defined per WHO classification as a SARS-CoV-2 infected person with “oxygen saturation of <90% on room air, and/or respiratory rate of >30 breaths/minute in adults and children >5 years old (or ≥60 breaths/minute in children <2 months old or ≥50 breaths/minute in children 2-11 months old or ≥40 breaths/minute in children 1–5 years old), and/or signs of severe respiratory distress (accessory muscle use and inability to complete full sentences, and, in children, very severe chest wall indrawing, grunting, central cyanosis, or presence of any other general danger signs)”.^22^ Detailed WHO criteria for classifying SARS-CoV-2 infection severity can be found in the WHO technical report.^22^

Critical COVID-19 disease was defined per WHO classification as a SARS-CoV-2 infected person with “acute respiratory distress syndrome, sepsis, septic shock, or other conditions that would normally require the provision of life sustaining therapies such as mechanical ventilation (invasive or non-invasive) or vasopressor therapy”.^22^ Detailed WHO criteria for classifying SARS-CoV-2 infection criticality can be found in the WHO technical report.^22^

COVID-19 death was defined per WHO classification as “a death resulting from a clinically compatible illness, in a probable or confirmed COVID-19 case, unless there is a clear alternative cause of death that cannot be related to COVID-19 disease (e.g. trauma). There should be no period of complete recovery from COVID-19 between illness and death. A death due to COVID-19 may not be attributed to another disease (e.g. cancer) and should be counted independently of preexisting conditions that are suspected of triggering a severe course of COVID-19”. Detailed WHO criteria for classifying COVID-19 death can be found in the WHO technical report.^23^

# ***Table S1*: Characteristics of matched cases and controls using the combined samples over all months of the studies investigating effectiveness against SARS-CoV-2 infection of previous SARS-CoV-2 infection, primary-series (two-dose) mRNA vaccination, and booster (third dose) mRNA vaccination.**

| **Characteristics** | **Study 1**  **Effectiveness of previous** **SARS-CoV-2 infection** | | | **Study 2**  **Effectiveness of primary-series vaccination** | | | **Study 3**  **Effectiveness of booster vaccination** | | |
| --- | --- | --- | --- | --- | --- | --- | --- | --- | --- |
|  | **Cases***  **n (%)** | **Controls***  **n (%)** | **SMD^†^** | **Cases^‡^**  **n (%)** | **Controls^‡^**  **n (%)** | **SMD^†^** | **Cases^‡^**  **n (%)** | **Controls^‡^**  **n (%)** | **SMD^†^** |
|  | **N=530,213** | **N=530,213** |  | **N=382,978** | **N=382,978** |  | **N=156,115** | **N=156,115** |  |
| Median age (IQR)—years | 32 (20-40) | 32 (20-40) | 0.00^§^ | 30 (15-39) | 30 (16-39) | 0.00^§^ | 27 (8-38) | 27 (8-38) | 0.00^§^ |
| Age—years |  |  |  |  |  |  |  |  |  |
| 0-9 years | 70,051 (13.2) | 70,051 (13.2) | 0.00 | 60,438 (15.8) | 60,438 (15.8) | 0.00 | 45,058 (28.9) | 45,058 (28.9) |  |
| 10-19 years | 62,049 (11.7) | 62,049 (11.7) |  | 51,444 (13.4) | 51,444 (13.4) |  | 19,896 (12.7) | 19,896 (12.7) |  |
| 20-29 years | 98,917 (18.7) | 98,917 (18.7) |  | 73,603 (19.2) | 73,603 (19.2) |  | 20,106 (12.9) | 20,106 (12.9) |  |
| 30-39 years | 160,078 (30.2) | 160,078 (30.2) |  | 110,005 (28.7) | 110,005 (28.7) |  | 35,226 (22.6) | 35,226 (22.6) |  |
| 40-49 years | 90,418 (17.1) | 90,418 (17.1) |  | 58,939 (15.4) | 58,939 (15.4) |  | 21,853 (14.0) | 21,853 (14.0) | 0.00 |
| 50-59 years | 34,878 (6.6) | 34,878 (6.6) |  | 20,557 (5.4) | 20,557 (5.4) |  | 9,483 (6.1) | 9,483 (6.1) |  |
| 60-69 years | 10,369 (2.0) | 10,369 (2.0) |  | 5,890 (1.5) | 5,890 (1.5) |  | 3,224 (2.1) | 3,224 (2.1) |  |
| 70-79 years | 2,558 (0.5) | 2,558 (0.5) |  | 1,522 (0.4) | 1,522 (0.4) |  | 914 (0.6) | 914 (0.6) |  |
| 80+ years | 895 (0.2) | 895 (0.2) |  | 580 (0.2) | 580 (0.2) |  | 355 (0.2) | 355 (0.2) |  |
| Sex |  |  |  |  |  |  |  |  |  |
| Male | 310,962 (58.7) | 310,962 (58.7) | 0.00 | 219,871 (57.4) | 219,871 (57.4) | 0.00 | 80,576 (51.6) | 80,576 (51.6) | 0.00 |
| Female | 219,251 (41.4) | 219,251 (41.4) |  | 163,107 (42.6) | 163,107 (42.6) |  | 75,539 (48.4) | 75,539 (48.4) |  |
| Nationality^¶^ |  |  |  |  |  |  |  |  |  |
| Bangladeshi | 25,361 (4.8) | 25,361 (4.8) | 0.00 | 17,700 (4.6) | 17,700 (4.6) | 0.00 | 3,029 (1.9) | 3,029 (1.9) | 0.00 |
| Egyptian | 32,437 (6.1) | 32,437 (6.1) |  | 23,224 (6.1) | 23,224 (6.1) |  | 10,584 (6.8) | 10,584 (6.8) |  |
| Filipino | 57,347 (10.8) | 57,347 (10.8) |  | 37,274 (9.7) | 37,274 (9.7) |  | 18,211 (11.7) | 18,211 (11.7) |  |
| Indian | 109,773 (20.7) | 109,773 (20.7) |  | 71,605 (18.7) | 71,605 (18.7) |  | 32,259 (20.7) | 32,259 (20.7) |  |
| Nepalese | 26,365 (5.0) | 26,365 (5.0) |  | 17,776 (4.6) | 17,776 (4.6) |  | 3,076 (2.0) | 3,076 (2.0) |  |
| Pakistani | 21,079 (4.0) | 21,079 (4.0) |  | 15,029 (3.9) | 15,029 (3.9) |  | 5,895 (3.8) | 5,895 (3.8) |  |
| Qatari | 125,512 (23.7) | 125,512 (23.7) |  | 98,159 (25.6) | 98,159 (25.6) |  | 39,767 (25.5) | 39,767 (25.5) |  |
| Sri Lankan | 12,600 (2.4) | 12,600 (2.4) |  | 8,913 (2.3) | 8,913 (2.3) |  | 2,549 (1.6) | 2,549 (1.6) |  |
| Sudanese | 15,096 (2.9) | 15,096 (2.9) |  | 11,181 (2.9) | 11,181 (2.9) |  | 3,965 (2.5) | 3,965 (2.5) |  |
| Other nationalities^**^ | 104,643 (19.7) | 104,643 (19.7) |  | 82,117 (21.4) | 82,117 (21.4) |  | 36,780 (23.6) | 36,780 (23.6) |  |
| Number of coexisting conditions |  |  |  |  |  |  |  |  |  |
| None | 401,897 (75.8) | 401,897 (75.8) | 0.00 | 296,231 (77.4) | 296,231 (77.4) | 0.00 | 117,363 (75.2) | 117,363 (75.2) | 0.00 |
| 1 | 73,842 (13.9) | 73,842 (13.9) |  | 53,034 (13.9) | 53,034 (13.9) |  | 23,603 (15.1) | 23,603 (15.1) |  |
| 2 | 27,745 (5.2) | 27,745 (5.2) |  | 18,300 (4.8) | 18,300 (4.8) |  | 7,758 (5.0) | 7,758 (5.0) |  |
| ≥3 | 26,729 (5.0) | 26,729 (5.0) |  | 15,413 (4.0) | 15,413 (4.0) |  | 7,391 (4.7) | 7,391 (4.7) |  |

Abbreviations: IQR, interquartile range, SARS-CoV-2, severe acute respiratory syndrome coronavirus 2, and SMD standardized mean difference.

^*^Cases and controls were matched exactly one-to-one by sex, 10-year age group, nationality, number of coexisting conditions, method of testing (PCR or rapid antigen), reason for SARS-CoV-2 testing, vaccine type, number of vaccine doses, and (by design) calendar month of testing.

^†^SMD is the difference in the mean of a covariate between groups divided by the pooled standard deviation. An SMD ≤0.1 indicates adequate matching.

^‡^Cases and controls were matched exactly one-to-one by sex, 10-year age group, nationality, number of coexisting conditions, method of testing (PCR or rapid antigen), reason for SARS-CoV-2 testing, status of most recent prior infection (no documented prior infection, documented pre-Omicron prior infection, or documented Omicron prior infection), and (by design) calendar month of testing.

^§^SMD is for the mean difference between groups divided by the pooled standard deviation.

^¶^Nationality groups were chosen to represent the most populous groups in Qatar.

^**^Each case in the “other nationalities” group was matched, in a one-to-one ratio to a control with identical nationality. This group comprises up to 137 other nationalities in cases and controls.

# ***Figure S1:* Flowchart illustrating the population selection process to investigate the effectiveness of previous SARS-CoV-2 infection against reinfection in Qatar in a given calendar month analysis.**





***Table S2*:** **Effectiveness of previous SARS-CoV-2 infection against reinfection in Qatar between July of 2020 and November of 2022.**

| **Calendar month** | **Cases**^*^ **(SARS-CoV-2-positive tests)** | | | **Controls**^*^ **(SARS-CoV-2-negative tests)** | | | **Effectiveness in %**  **(95% CI)**^†^ |
| --- | --- | --- | --- | --- | --- | --- | --- |
|  | **Median time between previous infection and SARS-CoV-2 test (IQR) in days** | **Previous infection (n)** | **No previous infection (n)** | **Median time between previous infection and SARS-CoV-2 test (IQR) in days** | **Previous infection (n)** | **No previous infection (n)** |  |
| **Jul, 2020** | 101 (97-103) | 5 | 20,713 | 94 (92-106) | 21 | 20,697 | 76.2 (36.9 to 91.0) |
| **Aug, 2020** | 102 (94-108) | 31 | 16,327 | 102 (95-113) | 130 | 16,228 | 76.7 (65.4 to 84.4) |
| **Sep, 2020** | 110 (98.5-124) | 88 | 14,003 | 115 (104-129) | 282 | 13,809 | 69.8 (61.4 to 76.3) |
| **Oct, 2020** | 127 (107-145) | 70 | 10,905 | 135 (115-151) | 342 | 10,633 | 80.0 (74.1 to 84.6) |
| **Nov, 2020** | 148.5 (121-175) | 30 | 6,014 | 158 (132-175) | 219 | 5,825 | 87.1 (80.9 to 91.3) |
| **Dec, 2020** | 174 (118-209) | 39 | 5,163 | 186 (154-209) | 273 | 4,929 | 86.3 (80.8 to 90.3) |
| **Jan, 2021** | 205.5 (123.5-230.5) | 64 | 10,312 | 204 (155-232) | 538 | 9,838 | 88.8 (85.3 to 91.4) |
| **Feb, 2021** | 235 (168-271) | 171 | 22,253 | 231 (170-260) | 1,263 | 21,161 | 87.3 (85.0 to 89.2) |
| **Mar, 2021** | 280 (230-306) | 555 | 55,637 | 262 (202-291) | 3,373 | 52,819 | 84.8 (83.3 to 86.2) |
| **Apr, 2021** | 297 (249.5-325) | 488 | 45,447 | 283 (215-315) | 2,677 | 43,258 | 82.9 (81.1 to 84.5) |
| **May, 2021** | 305.5 (158-347) | 106 | 8,376 | 308 (226-345) | 543 | 7,939 | 81.4 (76.9 to 85.0) |
| **Jun, 2021** | 140 (107-334) | 62 | 3,189 | 262 (128-359) | 198 | 3,053 | 70.5 (60.3 to 78.0) |
| **Jul, 2021** | 111 (99-302) | 55 | 2,718 | 246 (122-375) | 209 | 2,564 | 75.5 (66.6 to 82.0) |
| **Aug, 2021** | 174 (125-373) | 47 | 3,772 | 194 (140-360) | 430 | 3,389 | 90.3 (86.7 to 93.0) |
| **Sep, 2021** | 184 (161-321) | 50 | 2,217 | 217 (171-389) | 271 | 1,996 | 83.1 (76.8 to 87.7) |
| **Oct, 2021** | 207.5 (161-441.5) | 28 | 1,717 | 263 (195-426) | 209 | 1,536 | 90.0 (84.2 to 93.7) |
| **Nov, 2021** | 250 (218-355) | 74 | 4,403 | 272.5 (225-443) | 548 | 3,929 | 88.3 (84.8 to 91.0) |
| **Dec, 2021** | 326 (272-538) | 2,372 | 30,267 | 314 (258-503) | 4,452 | 28,187 | 51.0 (48.3 to 53.6) |
| **Jan, 2022** | 336 (284-516) | 12,872 | 134,056 | 326 (278-509) | 22,009 | 124,919 | 46.2 (44.9 to 47.5) |
| **Feb, 2022** | 349 (302-523) | 1,428 | 13,854 | 344 (299-530.5) | 2,156 | 13,126 | 38.0 (33.4 to 42.4) |
| **Mar, 2022** | 367 (331-548) | 191 | 2,336 | 357 (293-548) | 352 | 2,175 | 50.9 (40.6 to 59.5) |
| **Apr, 2022** | 380.5 (111.5-501.5) | 188 | 1,728 | 108 (97-365) | 534 | 1,382 | 74.4 (68.7 to 79.1) |
| **May, 2022** | 392 (141-494) | 361 | 2,790 | 132 (120-268) | 1,178 | 1,973 | 80.8 (77.6 to 83.5) |
| **Jun, 2022** | 425.5 (169-516.5) | 1,416 | 9,751 | 163 (150-400) | 3,925 | 7,242 | 76.6 (74.7 to 78.4) |
| **Jul, 2022** | 425 (195-521) | 3,617 | 17,453 | 195 (182-386) | 7,343 | 13,727 | 65.0 (63.2 to 66.8) |
| **Aug, 2022** | 302 (219-530) | 3,463 | 13,569 | 222 (210-455) | 5,645 | 11,387 | 52.6 (49.9 to 55.0) |
| **Sep, 2022** | 274 (251-567) | 4,729 | 14,738 | 256 (242-517) | 6,552 | 12,915 | 39.9 (37.0 to 42.7) |
| **Oct, 2022** | 290 (274-564) | 4,412 | 10,764 | 283 (269-519.5) | 5,504 | 9,672 | 31.8 (28.1 to 35.3) |
| **Nov, 2022** | 318 (304-575) | 2,641 | 6,088 | 312 (297-544) | 3,146 | 5,583 | 26.5 (21.3 to 31.4) |

Abbreviations: CI, confidence interval, IQR, interquartile range, PCR, polymerase chain reaction, and SARS-CoV-2, severe acute respiratory syndrome coronavirus 2.

^*^Cases and controls were matched exactly one-to-one by sex, 10-year age group, nationality, number of coexisting conditions, method of testing (PCR or rapid antigen), reason for SARS-CoV-2 testing, vaccine type, number of vaccine doses, and (by design) calendar month of testing.

^†^Effectiveness of previous infection in preventing reinfection was estimated using the test-negative, case–control study design.^24^

# ***Figure S2:* Flowchart illustrating the population selection process to investigate the effectiveness of primary-series (two-dose) mRNA vaccination against SARS-CoV-2 infection in Qatar in a given calendar month analysis.**

**

**

# ***Table S3:* Effectiveness of primary-series (two-dose) mRNA vaccination against SARS-CoV-2 infection in Qatar between February of 2021 and November of 2022.**

| **Calendar month** | **Cases**^*^ **(SARS-CoV-2-positive tests)** | | | **Controls**^*^ **(SARS-CoV-2-negative tests)** | | | **Effectiveness in %**  **(95% CI)**^†^ |
| --- | --- | --- | --- | --- | --- | --- | --- |
|  | **Median time between second vaccine dose and SARS-CoV-2 test (IQR) in days** | **Vaccinated (n)** | **Not vaccinated (n)** | **Median time between second vaccine dose and SARS-CoV-2 test (IQR) in days** | **Vaccinated (n)** | **Not vaccinated (n)** |  |
| **Feb, 2021** | 24 (16-31) | 35 | 21,863 | 22 (17-28) | 221 | 21,677 | 84.5 (77.8 to 89.2) |
| **Mar, 2021** | 34 (22-47) | 590 | 50,334 | 30 (20-43) | 2,394 | 48,530 | 81.8 (79.8 to 83.6) |
| **Apr, 2021** | 34 (23-53) | 2,043 | 39,210 | 28 (20-44) | 7,569 | 33,684 | 84.0 (83.0 to 85.0) |
| **May, 2021** | 48 (29-68.5) | 708 | 6,767 | 40 (26-59) | 2,016 | 5,459 | 80.9 (78.4 to 83.1) |
| **Jun, 2021** | 64 (35-92) | 481 | 2,549 | 49 (31-79) | 1,151 | 1,879 | 80.7 (77.2 to 83.7) |
| **Jul, 2021** | 107 (71-130) | 730 | 1,939 | 77 (51-114) | 1,258 | 1,411 | 76.2 (71.8 to 79.9) |
| **Aug, 2021** | 134 (94-155) | 1,638 | 2,162 | 113 (74-150) | 2,158 | 1,642 | 63.0 (57.8 to 67.5) |
| **Sep, 2021** | 157 (118-181) | 1,028 | 1,241 | 145 (97-176) | 1,316 | 953 | 61.9 (54.7 to 68.0) |
| **Oct, 2021** | 180 (135-210) | 761 | 1,014 | 163 (114-204) | 889 | 886 | 44.6 (32.8 to 54.4) |
| **Nov, 2021** | 209 (160-240) | 1,686 | 2,818 | 187 (133-230) | 2,096 | 2,408 | 52.7 (46.5 to 58.2) |
| **Dec, 2021** | 228 (187-265) | 20,139 | 10,896 | 213 (163-251) | 19,963 | 11,072 | -3.9 (-7.8 to 0.0) |
| **Jan, 2022** | 241 (192-279) | 77,910 | 51,637 | 237 (185-278) | 74,899 | 54,648 | -15.1 (-16.8 to -13.3) |
| **Feb, 2022** | 248 (199-299) | 6,095 | 7,786 | 253 (198-302) | 6,052 | 7,829 | -2.2 (-8.2 to 4.0) |
| **Mar, 2022** | 291 (236-347) | 1,025 | 1,060 | 282 (223-335) | 1,042 | 1,043 | 5.1 (-9.6 to 18.6) |
| **Apr, 2022** | 326.5 (267-373) | 822 | 723 | 322 (261-371) | 789 | 756 | -12.4 (-26.5 to 4.2) |
| **May, 2022** | 364 (289-412) | 1,206 | 1,092 | 344.5 (274-399.5) | 1,200 | 1,098 | -1.6 (-14.7 to 11.9) |
| **Jun, 2022** | 389 (321-439) | 4,063 | 3,362 | 381 (313-434) | 3,773 | 3,652 | -20.7 (-26.7 to -14.2) |
| **Jul, 2022** | 411 (346-460.5) | 7,528 | 7,237 | 409 (345-459) | 7,351 | 7,414 | -7.2 (-12.3 to -1.7) |
| **Aug, 2022** | 432 (360-483) | 5,589 | 6,399 | 428 (356-483) | 5,568 | 6,420 | -1.1 (-7.1 to 5.1) |
| **Sep, 2022** | 459 (393-510) | 6,990 | 6,186 | 460 (393-511) | 6,849 | 6,327 | -5.8 (-11.1 to -0.3) |
| **Oct, 2022** | 500 (434-549) | 5,672 | 4,338 | 498 (433-548) | 5,550 | 4,460 | -6.9 (-12.9 to -0.4) |
| **Nov, 2022** | 541 (486-585) | 3,064 | 2,562 | 533 (482-578) | 3,044 | 2,582 | -2.1 (-10.5 to 6.7) |

Abbreviations: CI, confidence interval, IQR, interquartile range, PCR, polymerase chain reaction, and SARS-CoV-2, severe acute respiratory syndrome coronavirus 2.

^*^Cases and controls were matched exactly one-to-one by sex, 10-year age group, nationality, number of coexisting conditions, method of testing (PCR or rapid antigen), reason for SARS-CoV-2 testing, status of most recent prior infection (no documented prior infection, documented pre-Omicron prior infection, or documented Omicron prior infection), and (by design) calendar month of testing.

^†^Vaccine effectiveness was estimated using the test-negative, case-control study design.^25,26^

# ***Figure S3:* Flowchart illustrating the population selection process to investigate the effectiveness of booster (third-dose) mRNA vaccination against SARS-CoV-2 infection in Qatar in a given calendar month analysis.**





# ***Table S4:*** **Effectiveness of booster (third-dose) mRNA vaccination against SARS-CoV-2 infection in Qatar between November of 2021 and November of 2022.**

| **Calendar month** | **Cases**^*^ **(SARS-CoV-2-positive tests)** | | | **Controls**^*^ **(SARS-CoV-2-negative tests)** | | | **Effectiveness in %**  **(95% CI)**^†^ |
| --- | --- | --- | --- | --- | --- | --- | --- |
|  | **Median time between third vaccine dose and SARS-CoV-2 test (IQR) in days** | **Vaccinated (n)** | **Not vaccinated (n)** | **Median time between third vaccine dose and SARS-CoV-2 test (IQR) in days** | **Vaccinated (n)** | **Not vaccinated (n)** |  |
| **Nov, 2021** | 27 (8.5-47) | 16 | 2,770 | 20 (12-34) | 60 | 2,726 | 83.0 (65.6 to 91.6) |
| **Dec, 2021** | 34 (20-52) | 1,948 | 10,541 | 26 (16-41) | 2,354 | 10,135 | 32.9 (26.7 to 38.5) |
| **Jan, 2022** | 43 (26-60) | 15,607 | 52,535 | 41 (24-57) | 16,996 | 51,146 | 18.1 (15.4 to 20.8) |
| **Feb, 2022** | 63 (38-82) | 1,591 | 7,719 | 60 (37-81) | 1,631 | 7,679 | 5.3 (-4.7 to 14.6) |
| **Mar, 2022** | 97.5 (64-116) | 484 | 1,051 | 80 (48-111) | 495 | 1,040 | 6.0 (-13.6 to 23.7) |
| **Apr, 2022** | 123 (85-144) | 422 | 715 | 101 (69-136) | 397 | 740 | -15.6 (-32.9 to 5.7) |
| **May, 2022** | 158 (125-178) | 864 | 1,090 | 135 (96-167) | 823 | 1,131 | -13.7 (-27.0 to 1.9) |
| **Jun, 2022** | 181 (148-204) | 3,827 | 3,343 | 164 (125-195) | 3,238 | 3,932 | -42.8 (-47.6 to -37.6) |
| **Jul, 2022** | 204 (170-231) | 6,110 | 7,214 | 193 (151-226) | 5,665 | 7,659 | -21.5 (-26.4 to -16.2) |
| **Aug, 2022** | 228 (190-257) | 4,952 | 6,361 | 215 (169-251) | 4,682 | 6,631 | -17.4 (-23.3 to -11.1) |
| **Sep, 2022** | 257 (217-288) | 6,017 | 6,140 | 245 (201-283) | 5,749 | 6,408 | -15.1 (-20.7 to -9.1) |
| **Oct, 2022** | 286 (245-316) | 4,979 | 4,305 | 278 (233-313) | 4,692 | 4,592 | -19.8 (-25.8 to -13.4) |
| **Nov, 2022** | 317 (278-349) | 2,973 | 2,541 | 307 (264-342) | 2,715 | 2,799 | -28.0 (-34.9 to -20.5) |

Abbreviations: CI, confidence interval, IQR, interquartile range, PCR, polymerase chain reaction, and SARS-CoV-2, severe acute respiratory syndrome coronavirus 2.

^*^Cases and controls were matched exactly one-to-one by sex, 10-year age group, nationality, number of coexisting conditions, method of testing (PCR or rapid antigen), reason for SARS-CoV-2 testing, status of most recent prior infection (no documented prior infection, documented pre-Omicron prior infection, or documented Omicron prior infection), and (by design) calendar month of testing.

^†^Vaccine effectiveness was estimated using the test-negative, case-control study design.^25,26^

# ***Table S5:* Effectiveness against severe, critical, or fatal COVID-19 of A) previous SARS-CoV-2 infection, B) primary-series (two-dose) mRNA vaccination, and C) booster (third-dose) mRNA vaccination, in Qatar, between July of 2020 and November of 2022.**

| **A) Effectiveness of previous SARS-CoV-2 infection** | | | | | | | |
| --- | --- | --- | --- | --- | --- | --- | --- |
| **Calendar month** | **Cases**^*^ **(SARS-CoV-2-positive tests)** | | | **Controls**^*^ **(SARS-CoV-2-negative tests)** | | | **Effectiveness in %**  **(95% CI)**^‡^ |
|  | **Median time between previous infection and SARS-CoV-2 test (IQR) in days** | **Previous infection (n)** | **No previous infection (n)** | **Median time between previous infection and SARS-CoV-2 test (IQR) in days** | **Previous infection (n)** | **No previous infection (n)** |  |
| **Jul-Dec, 2020** | 196 (196-196) | 1 | 1,582 | 138 (110-172) | 195 | 7,460 | 97.6 (82.8 to 99.7) |
| **Jan-Jun, 2021** | 280 (227-296) | 10 | 5,354 | 244 (191-288) | 1,777 | 23,479 | 97.6 (95.5 to 98.7) |
| **Jul-Dec, 2021** | 554.5 (514-595) | 2 | 360 | 237 (60-376) | 225 | 1,347 | 96.8 (87.0 to 99.2) |
| **Jan-Jun, 2022** | 294 (263.5-395.5) | 4 | 280 | 350 (277.5-508.5) | 232 | 967 | 94.1 (83.9 to 97.8) |
| **Jul-Nov, 2022** | 610 (550-670) | 2 | 29 | 217 (182.5-319) | 60 | 78 | 91.1 (60.5 to 98.0) |
| **B) Effectiveness of primary-series (two-dose) mRNA vaccination** | | | | | | | |
| **Calendar month** | **Cases**^†^ **(SARS-CoV-2-positive tests)** | | | **Controls**^†^ **(SARS-CoV-2-negative tests)** | | | **Effectiveness in %**  **(95% CI)**^‡^ |
|  | **Median time between second vaccine dose and SARS-CoV-2 test (IQR) in days** | **Vaccinated (n)** | **Not vaccinated (n)** | **Median time between second vaccine dose and SARS-CoV-2 test (IQR) in days** | **Vaccinated (n)** | **Not vaccinated (n)** |  |
| **Feb-Apr, 2021** | 49.5 (37-71) | 42 | 4,085 | 31 (21-47) | 2,749 | 16,974 | 95.4 (93.6 to 96.7) |
| **May-Jul, 2021** | 95 (79-107) | 25 | 386 | 64 (38-95) | 1,087 | 833 | 96.9 (94.8 to 98.1) |
| **Aug-Oct, 2021** | 189 (157.5-210.5) | 60 | 115 | 159 (116-190) | 649 | 156 | 91.4 (86.6 to 94.5) |
| **Nov, 2021-Jan, 2022** | 261 (202-304) | 161 | 206 | 242 (193-285) | 1,217 | 394 | 82.3 (76.6 to 86.6) |
| **Feb-Apr, 2022** | 254 (225-308) | 9 | 18 | 313.5 (275-356.5) | 76 | 35 | 81.4 (48.5 to 93.3) |
| **May-Jul, 2022** | 425 (314-447) | 3 | 8 | 431 (371-465) | 33 | 19 | 83.8 (14.1 to 96.9) |
| **Aug-Nov, 2022** | 453 (408-566) | 7 | 10 | 511 (442-564) | 39 | 32 | 53.1 (-41.3 to 87.1) |
| **C) Effectiveness of booster (third-dose) mRNA vaccination** | | | | | | | |
| **Calendar month** | **Cases**^†^ **(SARS-CoV-2-positive tests)** | | | **Controls**^†^ **(SARS-CoV-2-negative tests)** | | | **Effectiveness in %**  **(95% CI)**^‡^ |
|  | **Median time between third vaccine dose and SARS-CoV-2 test (IQR) in days** | **Vaccinated (n)** | **Not vaccinated (n)** | **Median time between third vaccine dose and SARS-CoV-2 test (IQR) in days** | **Vaccinated (n)** | **Not vaccinated (n)** |  |
| **Nov, 2021-Jan, 2022** | 84 (49-113) | 22 | 203 | 44 (23-75) | 460 | 508 | 95.0 (90.5 to 97.4) |
| **Feb-Apr, 2022** | 18.5 (9-28) | 2 | 17 | 96 (72-127) | 45 | 33 | 96.2 (69.6 to 99.5) |
| **May-Jul, 2022** | 225 (183-269) | 6 | 8 | 195 (139-218) | 33 | 27 | 43.6 (-51.4 to 84.5) |
| **Aug-Nov, 2022** | 328.5 (255.5-343) | 4 | 10 | 276 (219-306) | 30 | 24 | 90.4 (9.6 to 99.0) |

Abbreviations: CI, confidence interval, COVID-19, coronavirus disease 2019, IQR, interquartile range, PCR, polymerase chain reaction, and SARS-CoV-2, severe acute respiratory syndrome coronavirus 2.

^*^Cases and controls were matched exactly one-to-one by sex, 10-year age group, nationality, number of coexisting conditions, method of testing (PCR or rapid antigen), reason for SARS-CoV-2 testing, vaccine type, number of vaccine doses, and (by design) 6 calendar months of testing.

^†^Cases and controls were matched exactly one-to-one by sex, 10-year age group, nationality, number of coexisting conditions, method of testing (PCR or rapid antigen), reason for SARS-CoV-2 testing, status of most recent prior infection (no documented prior infection, documented pre-Omicron prior infection, or documented Omicron prior infection), and (by design) 6 calendar months of testing.

^‡^Effectiveness of previous infection in preventing reinfection and vaccine effectiveness were estimated using the test-negative, case–control study design.^24-26^

***References***

1. Altarawneh HN, Chemaitelly H, Ayoub HH, et al. Effects of Previous Infection and Vaccination on Symptomatic Omicron Infections. *N Engl J Med* 2022; **387**(1): 21-34.

2. Chemaitelly H, Tang P, Hasan MR, et al. Waning of BNT162b2 Vaccine Protection against SARS-CoV-2 Infection in Qatar. *N Engl J Med* 2021; **385**(24): e83.

3. Altarawneh HN, Chemaitelly H, Hasan MR, et al. Protection against the Omicron Variant from Previous SARS-CoV-2 Infection. *N Engl J Med* 2022; **386**(13): 1288-90.

4. Planning and Statistics Authority-State of Qatar. Qatar Monthly Statistics. Available from: <https://www.psa.gov.qa/en/pages/default.aspx>. Accessed on: May 26, 2020. 2020.

5. Abu-Raddad LJ, Chemaitelly H, Ayoub HH, et al. Characterizing the Qatar advanced-phase SARS-CoV-2 epidemic. *Sci Rep* 2021; **11**(1): 6233.

6. Abu-Raddad LJ, Chemaitelly H, Ayoub HH, et al. Effect of mRNA Vaccine Boosters against SARS-CoV-2 Omicron Infection in Qatar. *N Engl J Med* 2022; **386**(19): 1804-16.

7. Chemaitelly H, Faust JS, Krumholz H, et al. Short- and longer-term all-cause mortality among SARS-CoV-2- infected persons and the pull-forward phenomenon in Qatar. *medRxiv* 2023: 2023.01.29.23285152.

8. Chemaitelly H, Ayoub HH, Tang P, et al. Long-term COVID-19 booster effectiveness by infection history and clinical vulnerability and immune imprinting: a retrospective population-based cohort study. *Lancet Infect Dis* 2023.

9. Vogels C, Fauver J, Grubaugh N. Multiplexed RT-qPCR to screen for SARS-COV-2 B.1.1.7, B.1.351, and P.1 variants of concern V.3. dx.doi.org/10.17504/protocols.io.br9vm966. 2021; (June 6, 2021).

10. Abu-Raddad LJ, Chemaitelly H, Butt AA, National Study Group for Covid Vaccination. Effectiveness of the BNT162b2 Covid-19 Vaccine against the B.1.1.7 and B.1.351 Variants. *N Engl J Med* 2021; **385**(2): 187-9.

11. Chemaitelly H, Yassine HM, Benslimane FM, et al. mRNA-1273 COVID-19 vaccine effectiveness against the B.1.1.7 and B.1.351 variants and severe COVID-19 disease in Qatar. *Nat Med* 2021; **27**(9): 1614-21.

12. National Project of Surveillance for Variants of Concern and Viral Genome Sequencing. Qatar viral genome sequencing data. Data on randomly collected samples. <https://www.gisaid.org/phylodynamics/global/nextstrain/>. 2021. <https://www.gisaid.org/phylodynamics/global/nextstrain/>.

13. Benslimane FM, Al Khatib HA, Al-Jamal O, et al. One Year of SARS-CoV-2: Genomic Characterization of COVID-19 Outbreak in Qatar. *Front Cell Infect Microbiol* 2021; **11**: 768883.

14. Hasan MR, Kalikiri MKR, Mirza F, et al. Real-Time SARS-CoV-2 Genotyping by High-Throughput Multiplex PCR Reveals the Epidemiology of the Variants of Concern in Qatar. *Int J Infect Dis* 2021; **112**: 52-4.

15. Saththasivam J, El-Malah SS, Gomez TA, et al. COVID-19 (SARS-CoV-2) outbreak monitoring using wastewater-based epidemiology in Qatar. *Sci Total Environ* 2021; **774**: 145608.

16. El-Malah SS, Saththasivam J, Jabbar KA, et al. Application of human RNase P normalization for the realistic estimation of SARS-CoV-2 viral load in wastewater: A perspective from Qatar wastewater surveillance. *Environ Technol Innov* 2022; **27**: 102775.

17. Tang P, Hasan MR, Chemaitelly H, et al. BNT162b2 and mRNA-1273 COVID-19 vaccine effectiveness against the SARS-CoV-2 Delta variant in Qatar. *Nat Med* 2021; **27**(12): 2136-43.

18. Chemaitelly H, Ayoub HH, AlMukdad S, et al. Duration of mRNA vaccine protection against SARS-CoV-2 Omicron BA.1 and BA.2 subvariants in Qatar. *Nat Commun* 2022; **13**(1): 3082.

19. Qassim SH, Chemaitelly H, Ayoub HH, et al. Effects of BA.1/BA.2 subvariant, vaccination and prior infection on infectiousness of SARS-CoV-2 omicron infections. *J Travel Med* 2022; **29**(6).

20. Altarawneh HN, Chemaitelly H, Ayoub HH, et al. Protective Effect of Previous SARS-CoV-2 Infection against Omicron BA.4 and BA.5 Subvariants. *N Engl J Med* 2022; **387**(17): 1620-2.

21. Chemaitelly H, Tang P, Coyle P, et al. Protection against Reinfection with the Omicron BA.2.75 Subvariant. *N Engl J Med* 2023; **388**(7): 665-7.

22. World Health Organization (WHO). Living guidance for clinical management of COVID-19. Aavailable from: <https://www.who.int/publications/i/item/WHO-2019-nCoV-clinical-2021-2>. Accessed on: February 27, 2023. 2023.

23. World Health Organization (WHO). International Guidelines for Certification and Classification (Coding) of COVID-19 as Cause of Death. Available from: <https://www.who.int/publications/m/item/international-guidelines-for-certification-and-classification-(coding)-of-covid-19-as-cause-of-death>. Accessed on: February 27, 2023. 2023.

24. Ayoub HH, Tomy M, Chemaitelly H, et al. Estimating protection afforded by prior infection in preventing reinfection: Applying the test-negative study design. *medRxiv* 2022: 2022.01.02.22268622.

25. Jackson ML, Nelson JC. The test-negative design for estimating influenza vaccine effectiveness. *Vaccine* 2013; **31**(17): 2165-8.

26. Verani JR, Baqui AH, Broome CV, et al. Case-control vaccine effectiveness studies: Preparation, design, and enrollment of cases and controls. *Vaccine* 2017; **35**(25): 3295-302.
