# Supplementary material for: Population immunity of natural infection, primary-series vaccination, and booster vaccination in Qatar during the COVID-19 pandemic: an observational study
Source: eClinicalMedicine. 2023 Jul 20;62:102102. doi: 10.1016/j.eclinm.2023.102102 (PMC10393554; doi:10.1016/j.eclinm.2023.102102)
Supplement: STROBE checklist [file mmc2.docx]

# **STROBE checklist for case-control studies.**

|  | Item No | Recommendation | Main text page |
| --- | --- | --- | --- |
| **Title and abstract** | 1 | (*a*) Indicate the study’s design with a commonly used term in the title or the abstract | Abstract |
|  |  | (*b*) Provide in the abstract an informative and balanced summary of what was done and what was found | Abstract |
| Introduction | | |  |
| Background/rationale | 2 | Explain the scientific background and rationale for the investigation being reported | Introduction |
| Objectives | 3 | State specific objectives, including any prespecified hypotheses | Introduction |
| Methods | | |  |
| Study design | 4 | Present key elements of study design | Methods (‘Study design’) & Figures S1-S3 in Supplementary Appendix |
| Setting | 5 | Describe the setting, locations, and relevant dates, including periods of recruitment, exposure, follow-up, and data collection | Methods (‘Study population and data sources’ & ‘Study design’) & Section S1 in Supplementary Appendix |
| Participants | 6 | (*a*) Give the eligibility criteria, and the sources and methods of case ascertainment and control selection. Give the rationale for the choice of cases and controls | Methods (‘Study design’) & Figures S1-S3 in Supplementary Appendix |
|  |  | (*b*) For matched studies, give matching criteria and the number of controls per case |  |
| Variables | 7 | Clearly define all outcomes, exposures, predictors, potential confounders, and effect modifiers. Give diagnostic criteria, if applicable | Methods (‘Study design’ & ‘Statistical analysis’) & Sections S1-S3 in Supplementary Appendix |
| Data sources/ measurement | 8 | For each variable of interest, give sources of data and details of methods of assessment (measurement). Describe comparability of assessment methods if there is more than one group | Methods (‘Study population and data sources’, ‘Study design’ & ‘Statistical analysis’, paragraph 1) & Sections S1-S3 in Supplementary Appendix |
| Bias | 9 | Describe any efforts to address potential sources of bias | Methods (‘Study design’ & ‘Statistical analysis’) |
| Study size | 10 | Explain how the study size was arrived at | Methods (‘Study population and data sources’ & ‘Study design’) & Figures S1-S3 in Supplementary Appendix |
| Quantitative variables | 11 | Explain how quantitative variables were handled in the analyses. If applicable, describe which groupings were chosen and why | Methods (‘Study design’ & ‘Statistical analysis’) |
| Statistical methods | 12 | (*a*) Describe all statistical methods, including those used to control for confounding | Methods (‘Statistical analysis’) |
|  |  | (*b*) Describe any methods used to examine subgroups and interactions | Methods (‘Statistical analysis’) |
|  |  | (*c*) Explain how missing data were addressed | Not applicable, see Methods (‘Study population and data sources’) |
|  |  | (*d*) If applicable, explain how matching of cases and controls was addressed | Methods (‘Study design’& ‘Statistical analysis’) |
|  |  | (*e*) Describe any sensitivity analyses | Not applicable |
| Results | | |  |
| Participants | 13 | (a) Report numbers of individuals at each stage of study—eg numbers potentially eligible, examined for eligibility, confirmed eligible, included in the study, completing follow-up, and analysed | Figures S1-S3 & Tables S2-S5 in Supplementary Appendix |
|  |  | (b) Give reasons for non-participation at each stage |  |
|  |  | (c) Consider use of a flow diagram |  |
| Descriptive data | 14 | (a) Give characteristics of study participants (eg demographic, clinical, social) and information on exposures and potential confounders | Tables S1-S5 in Supplementary Appendix |
|  |  | (b) Indicate number of participants with missing data for each variable of interest | Not applicable, see Methods (‘Study population and data sources’) |
| Outcome data | 15 | Report numbers in each exposure category, or summary measures of exposure | Results, Figures 2 & 4, & Tables S2-S5 in Supplementary Appendix |
| Main results | 16 | (*a*) Give unadjusted estimates and, if applicable, confounder-adjusted estimates and their precision (eg, 95% confidence interval). Make clear which confounders were adjusted for and why they were included | Results, Figures 2 & 4, & Tables S2-S5 in Supplementary Appendix |
|  |  | (*b*) Report category boundaries when continuous variables were categorized | Figures 2 & 4, & Tables S2-S5 in Supplementary Appendix |
|  |  | (*c*) If relevant, consider translating estimates of relative risk into absolute risk for a meaningful time period | Not applicable |
| Other analyses | 17 | Report other analyses done—eg analyses of subgroups and interactions, and sensitivity analyses | Not applicable |
| Discussion | | |  |
| Key results | 18 | Summarise key results with reference to study objectives | Discussion, paragraphs 1-5 |
| Limitations | 19 | Discuss limitations of the study, taking into account sources of potential bias or imprecision. Discuss both direction and magnitude of any potential bias | Discussion, paragraphs 6-13 |
| Interpretation | 20 | Give a cautious overall interpretation of results considering objectives, limitations, multiplicity of analyses, results from similar studies, and other relevant evidence | Discussion, paragraph 14 |
| Generalisability | 21 | Discuss the generalisability (external validity) of the study results | Discussion, paragraphs 6-7 |
| Other information | | |  |
| Funding | 22 | Give the source of funding and the role of the funders for the present study and, if applicable, for the original study on which the present article is based | Acknowledgements |
